# A novel heterozygous *SIX1* missense mutation resulted in non-syndromic unilateral hearing loss

**DOI:** 10.3389/fgene.2022.1047230

**Published:** 2022-11-22

**Authors:** Ang Li, Siwen Liu, Peng Zhang, Xintong Hu, Guiying Li, Weiyue Gu, Yanfang Jiang

**Affiliations:** ^1^ Key Laboratory of Organ Regeneration and Transplantation of the Ministry of Education, Genetic Diagnosis Center, The First Hospital of Jilin University, Changchun, China; ^2^ Chigene (Beijing) Translational Medical Research Center Co., Ltd, Beijing, China

**Keywords:** non-syndromic unilateral hearing loss, SIX1 gene, whole exome sequencing, genetic counseling, novel mutation

## Abstract

Familial non-syndromic unilateral hearing loss (NS-UHL) is rare and its genetic etiology has not been clearly elucidated. This study aimed to identify the genetic cause of NS-UHL in a three-generation Chinese family. Detailed medical history consultation and clinical examination were conducted. Further, whole-exome sequencing (WES) was performed to identify the genetic etiology of the proband, and the variant was verified by Sanger sequencing. A novel missense mutation, c.533G>C (p.Arg178Thr), in the SIX homeobox 1 gene (*SIX1*) was identified in four patients and co-segregated with NS-UHL in a three-generation Chinese family as a dominant trait. Using bioinformatics analyses, we show that this novel mutation is pathogenic and affects the structure of SIX1 protein. These data suggest that mutations in *SIX1* gene are associated with NS-UHL. Our study added the NS-UHL phenotype associated with *SIX1*, and thereby improving the genetic counseling provided to individuals with *SIX1* mutations.

## 1 Introduction

Unilateral hearing loss (UHL) is estimated to occur in 0.83 per 1,000 newborns (Prieve et al., 2000). As with bilateral hearing loss, UHL can severely affect the individuals’ lives. The etiology of approximately 35%–60% of UHL cases remains unknown (Kinney, 1953; Everberg, 1960a; Brookhouser et al., 1991). The most commonly etiologies of UHL include sequelae of bacterial meningitis, complication of viral infection, prenatal or perinatal problems, head trauma, and even genetic alterations (van Wieringen et al., 2019). With the rapid development of next-generation sequencing, genetic alterations have been identified in bilateral hearing loss, and the gene mutation spectrum has been constantly improving (https://hereditaryhearingloss.org/). However, the genetic alterations accounting for UHL have not been clearly elucidated. There is no doubt that identifying the causal genes will benefit patients in diagnosis, genetic counseling, and drug development.

UHL is often dismissed as sporadic or environmental, and the genetic basis has not been explored in depth (Dodson et al., 2012). It can be inherited as part of Pendred syndrome (PS [MIM: 274600]) or Waardenburg syndrome (WS [MIM: PS193500]). Among UHL, familial non-syndromic unilateral hearing loss (NS-UHL) (MIM:125000) is rare; only a few families are described in the literature (Everberg, 1957; 1960b; Dikkers et al., 2005). The gene *KITLG* has been linked to NS-UHL (Zazo Seco et al., 2015). Additionally, Dodson et al. included 34 patients with NS-UHL in a national hereditary deafness repository proved that mutations in the *TECTA* and *COCH* genes that might be causally related to the NS-UHL (Dodson et al., 2012). However, the molecular basis for other genetic causes of NS-UHL remains an important gap in current knowledge.

The absence of mutational hot genes or spots in NS-UHL hindered mutational analysis with gene panels (Dodson et al., 2012; Zazo Seco et al., 2015). Whole-exome sequencing (WES), combined with validated bioinformatics tools have facilitated the detection of variants, particularly the single nucleotide variants (SNVs) and the small insertions and deletions (InDels). Moreover, WES is being widely used in clinical practice due to its high diagnostic yields, low cost, and excellent advantages in novel genes analysis and subsequent investigation.

In this study, we performed WES to identify the genetic cause of autosomal-dominant NS-UHL in a three-generation Chinese Han pedigree. A novel missense mutation, c.533G>C (p.Arg178Thr), in the *SIX1* gene was found to co-segregate with NS-UHL in this family. Our study added the NS-UHL phenotype associated with *SIX1*, and thereby improving the genetic counseling provided to individuals with *SIX1* mutations.

## 2 Materials and methods

### 2.1 Family members

**FIGURE 1:**
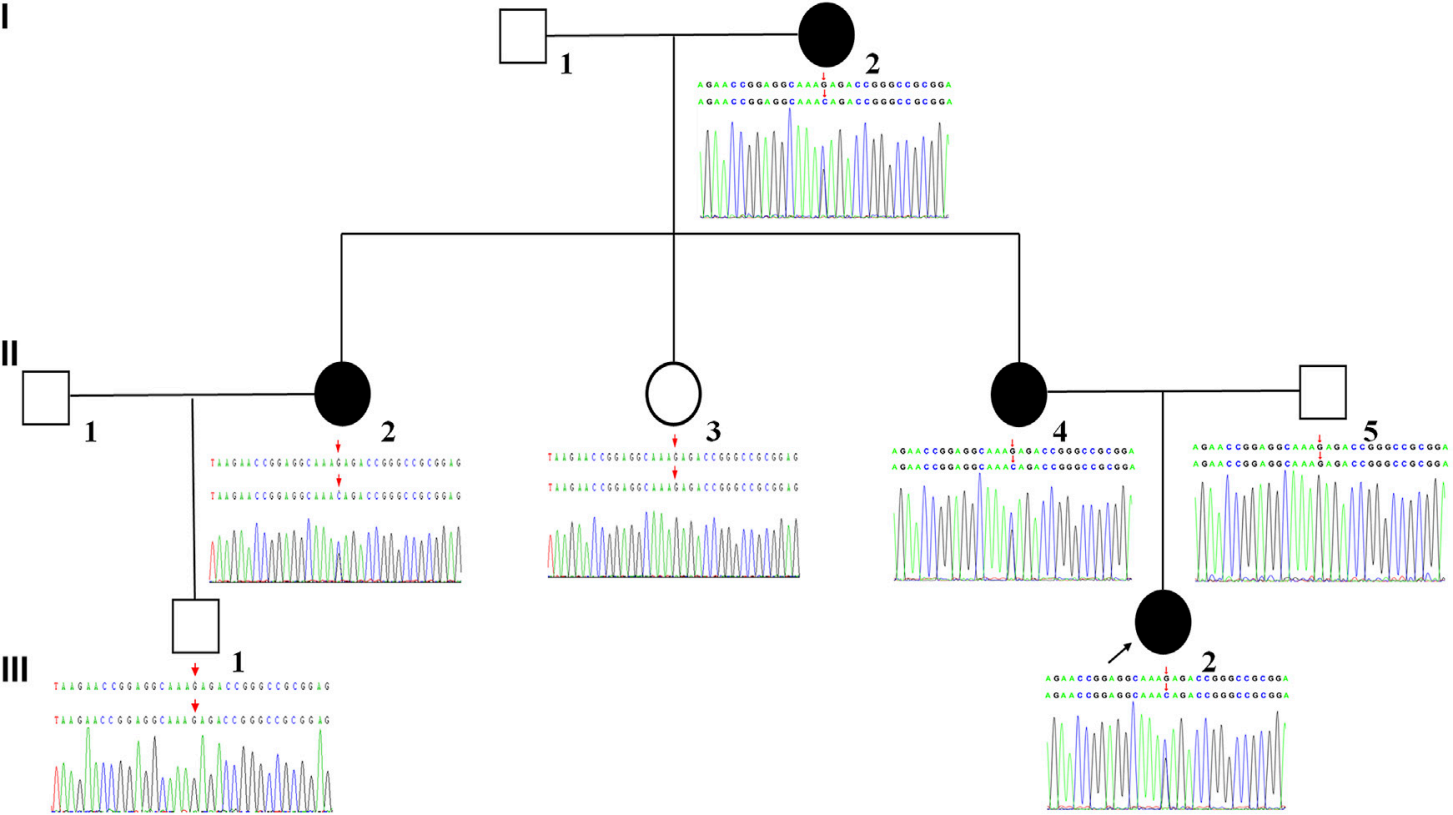
Pedigree of the family affected by NS-UHL. White symbols indicate normal individuals. The filled black symbol denotes the individual diagnosed with NS-UHL. The arrow indicates the proband of the family.

The proband (III-2) was a 7-year-old girl diagnosed with severe sensorineural hearing loss in the right ear at birth. The healthy side has normal hearing spectrum. Blood routine, urine routine, liver function and renal function showed normal parameters. The patient had no history of drug intoxication, trauma, or infection. Additionally, there was a family history of unilateral sensorineural hearing loss in her pedigree. Patients I-2, II-2, and II-4 also showed unilateral sensorineural hearing loss (Figure 1). This study was approved by the Ethics Committee of the First Hospital of Jilin University. All participants provided written informed consent.

### 2.2 Whole exome sequencing

Genomic DNA was isolated from whole blood of all available family members using the QIAamp DNA Blood MiNi Kit (Qiagen, Germany) according to the manufacturer’s instructions. We performed genetic testing using WES technology on the proband (III-2). Whole-exome capture was xGen Exome Research Panel v2.0 (IDT, Iowa, United States). High-throughput sequencing was performed on the DNBSEQT7 (BGI, China) platform. Average sequencing depth of WES was 162.75 × with an average of 97.7% of reads covered at a depth of at least 20×. For SIX1 gene, the average depth of sequencing was 167.56×. Raw data sequenced by WES were processed by using fastp (https://github.com/OpenGene/fastp) to remove adapters and filtering low-quality reads. High-quality reads were aligned to the GRCh37/hg19 reference genome using Burrows-Wheeler Aligner (BWA, https://github.com/lh3/bwa). Base quality score recalibration and calling variant were performed using the Genome Analysis Toolkit (GATK, http://www.broadinstitute.org/gatk/). The pipeline of variants analysis is as follows. According to the sequence depth and variant quality, the high-quality and reliable variants were obtained. Variants were then annotated with minor allele frequencies (MAFs) databases (genomAD, ESP, 1,000 genomes, EXAC, dbSNP databases). The Effect of the variant on gene product was predicted by using bioinformatics softwares such as Provean (http://provean.jcvi.org/genome_submit_2.php?species=human), REVEL (https://sites.google.com/site/revelgenomics/), SIFT (http://sift.jcvi.org/), GERP (http://mendel.stanford.edu/sidowlab/downloads/gerp/index.html) and phastCons (https://varianttools.sourceforge.net/Annotation/PhastCons). According to the American College of Medical Genetics criteria (ACMG), the identified variants were classified as pathogenic, likely pathogenic, benign, likely benign, or of uncertain significance (Richards et al., 2015; Harrison et al., 2019). Online Mendelian Inheritance in Man (OMIM), Human Gene Mutation Database (HGMD), and ClinVar databases were then used as conferences of pathogenicity of every variant. Finally, the most possible pathogenic genes were identified from the screened deleterious variants combining disease correlation and clinical phenotype. The variants filtering criteria was shown in Supplementary Table S1.

### 2.3 Sanger sequencing

We designed specific primers (forward 5′-CGC​CCA​CCG​CCA​AGT​TCC​GAC​TCC-3′ and reverse 5′-CCC​GAC​ACT​CAC​ATC​CCA​GAG​AAA​CCC​AC-3′) based on the variant loci detected by next-generation sequencing (NGS). DNA isolated from all available family was used as a template for PCR amplification on HEMA 9600 PCR sequencer using the specific primers for *SIX1* gene. Sanger sequencing was further performed using the ABI 3730XL sequencer (Applied Biosystems). Co-segregation was analyzed in all available members.

### 2.4 Protein conserved prediction and 3D structure prediction

We performed multiple sequence alignments using MUSCLE (https://www.ebi.ac.uk/Tools/msa/muscle/) and prepared model diagrams of protein domain using Illustrator for Biological Sequences V1.0 (IBS). The protein sequence encoded by the transcript (NM_005982.4) and the X-ray crystal structure (PDB ID 4EGC) were used for SWISS-MODEL with 100% sequence similarity, and PyMOL (PyMol Molecular Graphics System, Schrödinger, LLC) was used to visualize the model by referring to Version 2.1.0.

## 3 Results

### 3.1 *SIX1* gene mutations and sanger sequencing validation

A novel heterozygous missense variant in *SIX1* gene was detected in the proband (III-2) by using WES. The missense variants *SIX1* c.533G>C resulted in a threonine substitution. The frequency of the variant was absent from control database (gnomAD database, ESP database, 1,000 genomes database, EXAC database, dbSNP database). The variant were then validated in all available members by Sanger sequencing and revealed that the variant was maternally inherited. The family members (I-2, II-2, and II-4) with NS-UHL shared the same variant with the proband. Whereas, other family members (II-3, II-5, and III-1) without NS-UHL did not have any variants detected by Sanger sequencing. We verified that NS-UHL cosegregated with the *SIX1* c.533G>C mutation (Figure 1).

### 3.2 Bioinformatics analyses of the identified mutation

**FIGURE 2:**
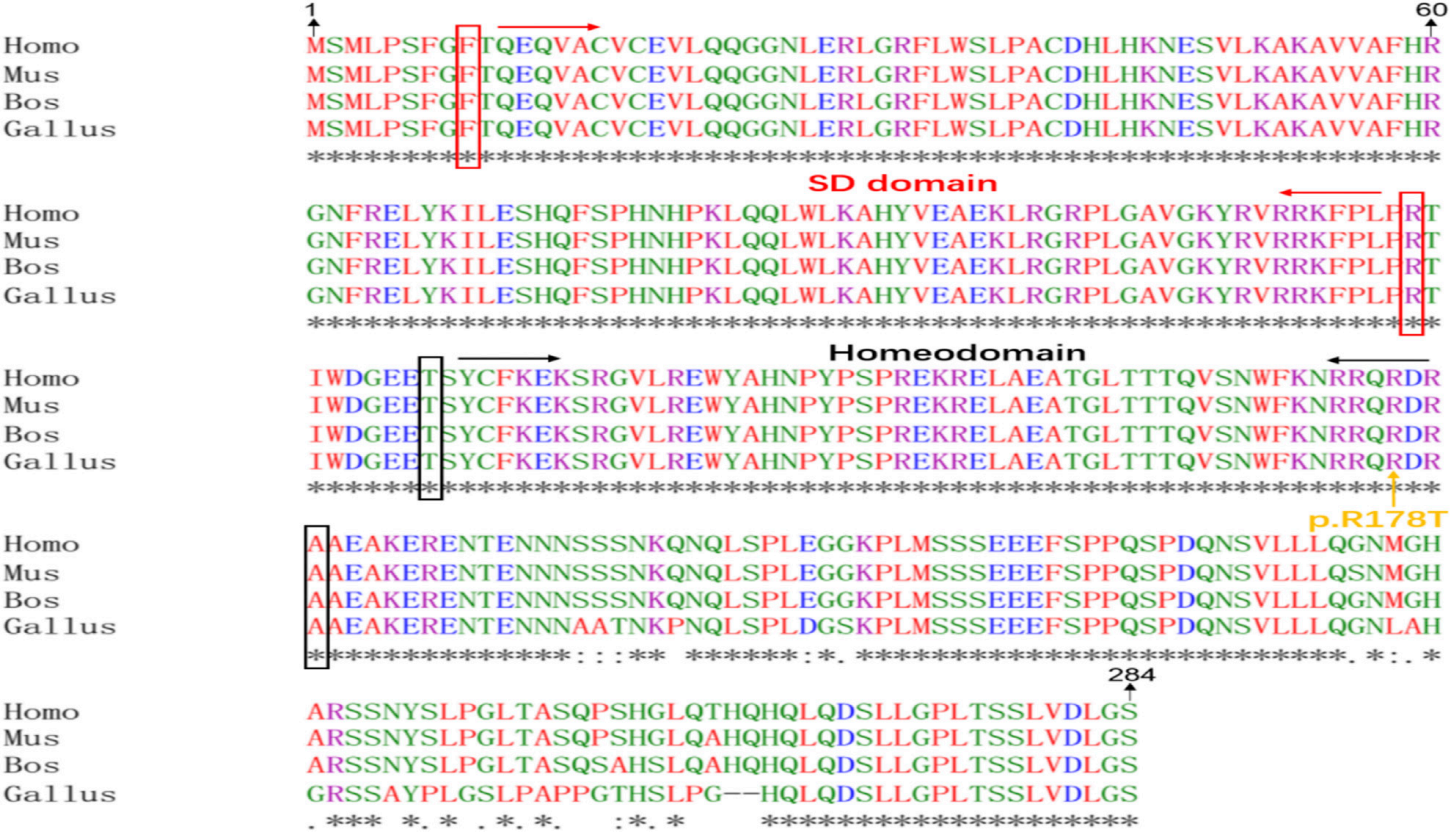
Alignment of SIX1 protein sequences from different species. Homo:*Homo sapiens* (NM_005982.4), Mus: *Mus musculus* (NM_009189.3), Bos:Bos Taurus (XM_002691019.6), Gallus:Gallus gallus (NM_001044685.2). Red: amino acid containing non-polar and hydrophobic R group, Green: amino acid containing polar and neutral R group, Blue: amino acid containing acidic R group, Purple: amino acid containing basic R group. “*”: a completely consistent residue, “.”: residues with weak and similar properties, “:”: residues with very similar properties.

**FIGURE 3:**
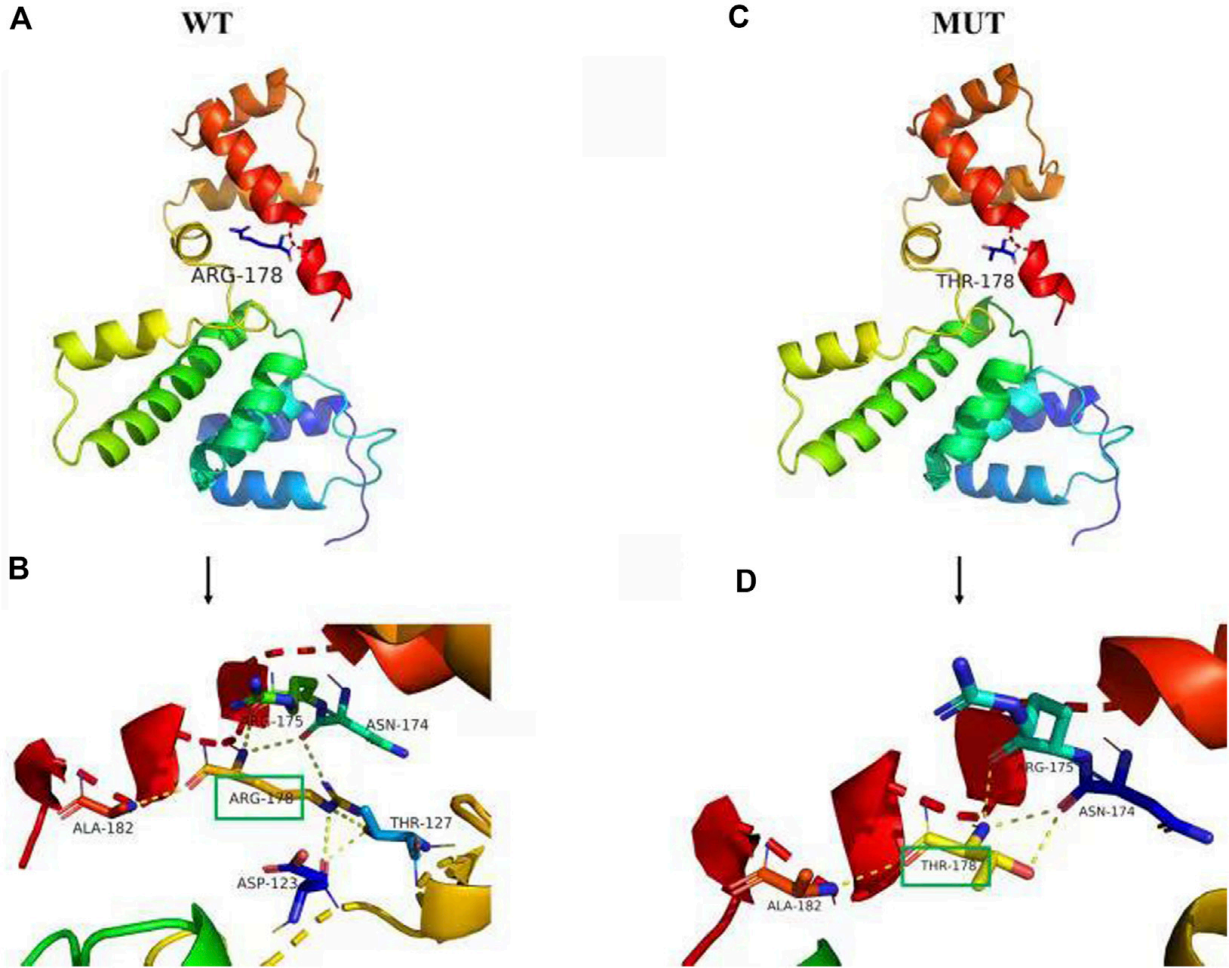
Prediction of the tertiary structure of SIX1 protein. The novel variant of the *SIX1* gene is c.533 G>C, p.R178T (p.Arg178Thr), **(A)** is the 3D overall picture of wild-type (WT) SIX1 protein: the 178th amino acid is ARG. **(B)** is the 3D partial enlarged view of WT SIX1 protein. The 178ARG respectively form a hydrogen bond with the 123ASP, 127THR, 175 ARG, and 182 ALA, and 178ARG form two hydrogen bonds with 174ASN, together to maintain the stability of the protein structure. **(C)** is the 3D overall picture of mutant (MUT) SIX1 protein: the 178th amino acid is mutated to THR. **(D)** is the 3D partial enlarged view of mutant SIX1 protein. No hydrogen bond interaction existed between the 178THR and 123ASP, 127THR.

All scores generated by different prediction softwares suggested deleterious effect: Provean (score -4.92), REVEL (score 0.947), SIFT (score 0.0) GERP (score 5.96) and phastCons (score 1.0). The amino acids in SIX1 protein, including arginine at position 178 (p.Arg178), are conserved across vertebrates from human to Gallus (Figure 2) and are located in the highly conserved homologous domain (HD) (Figure 2). Using PyMOL software to analyze the 3D structure of wild-type and mutant SIX1 proteins, the results (Figure 3) showed that compared with the wild-type, the c.533G>C variant substituted the neutral amino acid threonine for basic amino acid arginine at position 178 of the protein (p.Arg178Thr). In addition, compared with wild-type, the hydrogen bond between the 178th amino acid and the 123th and 127th amino acid of protein disappeared after mutation, which might affect the protein structure and function. According to the ACMG criteria, this mutation is pathogenic (Richards et al., 2015).

## 4 Discussion

UHL is often caused by acquired and unnoticed trauma, infection or other factors (van Wieringen et al., 2019). About 35%–60% of UHL cases do not receive an etiological diagnosis, which has stalled treatment for this disease. With the rapid development of next-generation sequencing, genetic alterations have also been identified as a cause of UHL. Previously, UHL frequently inherited as part of Pendred syndrome or Waardenburg syndrome that has been reported continuously. However, NS-UHL is rarely reported. Here, we reported a novel missense mutation in *SIX1* gene and provide evidence that this pathogenic mutation may be responsible for dominantly inherited NS-UHL.

The *SIX1* gene is located at chromosome 14q23.1 and is composed of an amino terminus, SIX domain (SD), homologous domain (HD) and a carboxyl terminus. The SD and HD domain were highly conserved. The *SIX1* gene plays an essential role in the development of several organs, including kidney, muscle and inner ear (Wu et al., 2014; Shah et al., 2020). According to the HGMD, only 24 pathogenic variants of the *SIX1* gene have been reported in the literature. Individuals carrying a heterozygous *SIX1* mutation were reported to have a very different clinical outcomes, ranging from being non-syndromic bilateral hearing loss to Branchio-otic (BO) syndrome and to Branchio-otic-renal (BOR) syndrome (Salam et al., 2000; Ruf et al., 2004; Mosrati et al., 2011).

Salam et al. demonstrated that heterozygous mutation in the *SIX1* gene associated with prelingual bilateral symmetric hearing loss (Salam et al., 2000). Mosrati et al. also proved that dominant mutation in *SIX1* result in only auditory defects in humans (Mosrati et al., 2011). Differently, Ruf and Kumar et al. reported *SIX1* mutations associated with branchio-otic syndrome or branchio-oto-renal syndrome (Kumar et al., 2000; Ruf et al., 2003; Ruf et al., 2004). Thus, the clinical phenotype associated with *SIX1* gene mutation need to be further enriched. In this study, we identified a novel missense mutation, *SIX1* c.533G>C in a three-generation Chinese Han pedigree and co-segregate with NS-UHL. Our study added the NS-UHL phenotype associated with *SIX1*. However, the effect of *SIX1* gene on the clinical phenotype has not been fully elucidated, and expanding the hearing loss family sample sizes is essential.

## 5 Concluding remarks

In conclusion, a novel missense mutation, c.533G>C (p.Arg178Thr), in the *SIX1* gene was identified in a Chinese Han family with NS-UHL. To the best of our knowledge, this is the first report on the association of the *SIX1* gene with NS-UHL. The mechanisms underlying this unilateral hearing loss is not clearly understood, further functional studies of the *SIX1* mutations and the application of *in vivo* models with genetic deficiency are warranted. This finding not only enriches the spectrum of diseases caused by *SIX1* gene, but also provides a new focus for genetic counseling.

## Data Availability

The original contributions presented in the study are publicly available. This data can be found here: https://www.ncbi.nlm.nih.gov/clinvar/ ,SCV002575099
