# Supplementary material for: A novel heterozygous SIX1 missense mutation resulted in non-syndromic unilateral hearing loss
Source: Front Genet. 2022 Nov 22;13:1047230. doi: 10.3389/fgene.2022.1047230 (PMC9723219; doi:10.3389/fgene.2022.1047230)
Supplement: Supplementary file 1 [file Table1.DOCX]

**Supplementary Table 1 Variants Filtering Criteria**

| **Filtering step** | **Filtering conditions** | **SNVs** | **Indels** |
| --- | --- | --- | --- |
| step 1 | All variants in the patient were obtained | 53491 | 7629 |
| step 2 | The following variants were filtered by basic filtering: deeper intronic region (intron >30 bp), low-quality SNP data (variation frequency <0.2 or sequencing depth <4x or quality value < 35), low frequency simple sequence repeat (SSR)/Indel (SSR>7 and AF<0.3) and indels  with length> 50 bp | 38738 | 4656 |
| step 3 | ACMG classification of variants: pathogenic (P) and likely pathogenic (LP) | 17 | 12 |
| step 4 | Support genetic pathogenesis | 11 | 3 |
| step 5 | Screened deleterious variants combining disease correlation and clinical phenotype | 1 |  |
